# Atrial High-Rate Episodes in Elderly Patients: The Anticoagulation Therapy Dilemma

**DOI:** 10.3390/jcm13123566

**Published:** 2024-06-18

**Authors:** Lorenzo Pimpini, Leonardo Biscetti, Giulia Matacchione, Cinzia Giammarchi, Michelangela Barbieri, Roberto Antonicelli

**Affiliations:** 1 Cardiology Unit, IRCCS INRCA, Via della Montagnola 81, 60127 Ancona, Italy; r.antonicelli@inrca.it; 2 Neurology Unit, IRCCS INRCA, 60127 Ancona, Italy; l.biscetti@inrca.it; 3 Clinic of Laboratory and Precision Medicine, IRCCS INRCA, 60121 Ancona, Italy; g.matacchione@inrca.it; 4 Scientific Direction, IRCCS INRCA, 60127 Ancona, Italy; c.giammarchi@inrca.it; 5 Department of Advanced Medical and Surgical Sciences, University of Campania “Luigi Vanvitelli”, 80138 Naples, Italy; michelangela.barbieri@unicampania.it

**Keywords:** atrial fibrillation, elderly subjects, atrial high-rate episodes, anticoagulant therapy

## Abstract

Atrial fibrillation (AF) has been associated with higher morbidity and mortality rates, especially in older patients. Subclinical atrial fibrillation (SCAF) is defined as the presence of atrial high-rate episodes (AHREs) > 190 bpm for 10 consecutive beats > 6 min and <24 h, as detected by cardiac implanted electronic devices (CIEDs). The selection of eligible patients for anticoagulation therapy among elderly individuals with AHREs detected through CIEDs remains a contentious issue. The meta-analysis of ARTESiA and NOAH-AFNET 6 clinical trials revealed that taking Edoxaban or Apixaban as oral anticoagulation therapy can reduce the risk of stroke by approximately 32% while increasing the risk of major bleeding by approximately 62%. However, it is still unclear which are, among patients with SCAF, those who can take the highest net clinical benefit from anticoagulant therapy. The present review summarizes the current evidence on this intriguing issue and suggests strategies to try to better stratify the risk of stroke and systemic embolism in patients with AHREs. We propose incorporating some parameters including chronic kidney disease (CKD), obesity, enlarged left atrial volume, the efficacy in blood pressure management, and frailty into the traditional CHA_2_DS_2_-VASc score. Future trials will be needed to verify the clinical usefulness of the proposed prognostic score mainly in the view of a personalized therapeutic approach in patients with SCAF.

## 1. Introduction

Atrial fibrillation (AF) is the most common sustained arrhythmia among various atrial tachyarrhythmias (AT). It is associated with a fivefold increase in stroke risk and a twofold rise in mortality [1,2].

AF is a supraventricular tachyarrhythmia with uncoordinated atrial electrical activation and ineffective atrial contraction. Clinical diagnosis of AF requires a surface electrocardiogram (ECG).

AF is characterized by electrocardiographic traits such as irregular R-R intervals, a lack of distinct repeating P waves, and irregular atrial activations [3].

AF is more prevalent in older individuals compared to younger ones. Among people aged 80 and above, the prevalence of AF is approximately 10%, and the estimated incidence is 50 per 1000 person/years in white women and 65 per 1000 person/years in white men [4]. However, these data are likely to be underestimated since AF, as well as other AT including atrial flutter, is often asymptomatic or pauci-symptomatic [5].

AF’s subtle symptoms can often result in a significantly delayed diagnosis, which can be extremely dangerous as AF patients are at a high risk of stroke, which increases with their cardiovascular risk level. Administering anticoagulation therapy promptly can significantly reduce this risk [6].

The CHA_2_DS_2_-VASc score is the primary method for assessing stroke risk in patients with AF, taking into account age, sex, and other vascular risk factors. This includes congestive heart failure (1 point), hypertension (1 point), age > 75 years old (2 points) or between 65 and 75 years old (1 point), diabetes history (1 point), previous stroke or transient ischemic attack or thromboembolism (2 points), vascular disease (1 point), and female sex (1 point).

The latest 2023 guidelines of the American Cardiovascular Care (ACC)/American Heart Association (AHA) [7] with respect to the previous 2020 guidelines of the European Society of Cardiology (ESC) [3] have renewed the recommendation to prescribe anticoagulant drugs to AF patients with an intermediate or high risk of stroke. An intermediate risk is defined as an annual stroke risk of more than 1% but less than 2%, which is equivalent to a CHA_2_DS_2_-VASc score of 1 in men and 2 in women. A high risk is when the annual stroke risk exceeds 2%, equivalent to a CHA_2_DS_2_-VASc score of 2 or higher in men and 3 or higher in women.

The ACC/AHA/ACCP/HRS 2023 guidelines [7] have upgraded the strength of recommendation for intermediate-risk patients from class IIa level B to class IIa level A. The highest level (class Ia level A) remains unchanged for high-risk patients as compared to the previous ESC 2020 guidelines. Studies conducted between 2002 and 2016 found that the occurrence of atrial fibrillation in patients with CIEDs ranged from 30% to 60% (Table 1).

Recognizing the significant relationship between AF and stroke, the use of cardiac implanted electronic devices (CIEDs) has become a common approach for screening and identifying potential stroke causes following an embolic stroke of undetermined source (ESUS), with the ultimate goal of promoting prompt detection of AF.

Subclinical atrial fibrillation (SCAF) is defined as atrial high-rate episodes (AHREs) lasting between 6 min and 24 h, with a heart rate exceeding 190 beats per minute for at least 10 consecutive beats, and showing little to no symptoms. This has been linked to a higher risk of stroke from thromboembolism and typically occurs before the onset of clinical AF [8]. The SCAF/AHRE definition is still not universally accepted since there is a relevant uncertainty about the threshold to be adopted.

**Figure 1:**
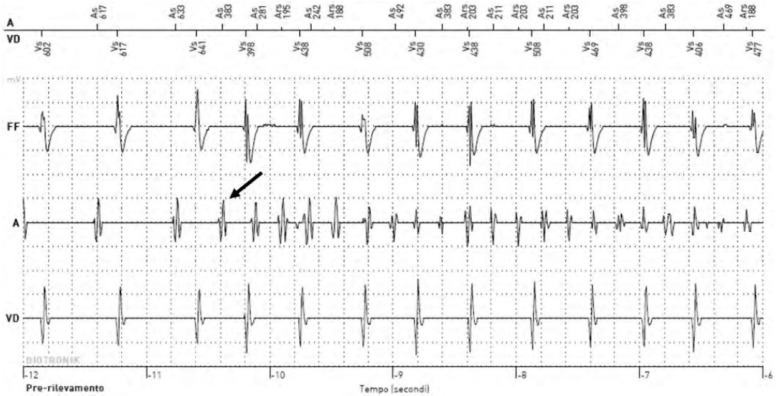
Example of an electrogram of an atrial high-rate episode detected through a CIED. The arrow indicates the detection of the SCAF.

SCAF is detected by all CIEDs that have remote monitoring such as permanent pacemakers, implantable cardioverter defibrillators (ICDs), implantable cardiac loop recorders, and cardiac resynchronization therapy (CRT) devices (Figure 1) [9].

The selection of eligible patients for anticoagulation therapy among elderly individuals with AHREs detected by CIEDs remains a quite controversial issue.

In this review, after analyzing the existing literature, we aim to highlight the importance of creating innovative tools that can be tested in future randomized clinical trials to improve the current clinical management of patients with AHREs.

Going into further detail, as deeply discussed later in this manuscript, the available evidence suggests that, at the general level, anticoagulant therapy can reduce the ischemic risk and increase the hemorrhagic risk in patients with SCAF, but, at the individual level, the net clinical benefit of anticoagulation is not always clear.

In light of this, we propose new scoring criteria that consider comorbidities associated with an increased risk of clinical AF and stroke. We believe it is important to assess the suitability of these scores in the SCAF context for potential integration into future clinical practice, along with scores that address the risk of bleeding. Furthermore, considering the limited evidence currently available, we discuss the potential implications of frailty status when determining the initiation of anticoagulant treatment in individuals with SCAF.

## 2. What Is the Appropriate Threshold for Detecting Atrial High-Rate Episodes?

In a meta-analysis of 54 studies including a total of 72,784 patients with CIEDs, the pooled prevalence of SCAF was 28.1%, with high heterogeneity among studies. Older age, history of AF, hypertension, higher CHA_2_DS_2_-VASC score, chronic heart failure (CHF), and stroke/TIA were all associated with SCAF occurrence, while male sex, body mass index, diabetes, and coronary artery disease were not associated with SCAF [10].

During a 2.5-year follow-up, the presence of AHREs was found to be linked with a higher likelihood of developing systemic embolism or ischemic stroke and clinical AF [11].

Evidence from another study revealed that individuals with single AHREs lasting at least 30 s and a total cumulative duration of AHRE exceeding 24 h are more likely to experience a stroke or systemic embolism [12].

However, the precise contribution of SCAF to the increased risk of stroke remains unclear. In recent years, several studies have evaluated the potential risk of thromboembolic stroke in patients with SCAF phases (ranging from 5 min to 24 h) detected in implanted devices. Table 2 summarizes the results.

The Ancillary MOST study, which began in 2003 [8], considered a minimum of 5 min of SCAF as a threshold for clinical significance. During the following decade, SCAF events with durations from 5 min to 24 h were examined, ultimately confirming the clinical significance of AHREs lasting more than 6 min [11].

All studies on AHREs have used arbitrary cut-points for the SCAF duration threshold, leading to uncertainty regarding the minimum duration of SCAF that can heighten the risk of thromboembolic events. Furthermore, it is noteworthy that there is a correlation between the chosen threshold and the risk of false-positive results. Specifically, in the ASSERT study, approximately 17.3% of AHREs recorded by CIEDs lasting longer than 6 min were false positives. When the duration threshold was lessened from 24 h to 6 h and to 30 min, false positives increased to 1.8%, 3.3%, and 6.8%, respectively [11].

False-positive AHREs can occur during CIED follow-up due to myopotential oversensing, electromagnetic interferences, lead atrial failure, and ineffective atrial pacing (non-reentrant ventriculo-atrial synchrony). Additionally, CIEDs can cause inaccurate results, such as failure to diagnose AF, leading to false-negative AHRE readings. This is because AHREs can be attributed to either true atrial under sensing, such as missed detection of AF due to weak signals, or functional atrial under sensing, where AF potentials align with atrial blanking periods [13].

As a result, taking into consideration the significant possibility of false-positive outcomes, short bouts of AHREs lasting less than 5 min are often disregarded in the majority of clinical studies due to their potential irrelevance.

In order to establish the link between SCAF duration and stroke risk in older patients with CIEDs, a case-crossover study was conducted. The study included 891 participants (64.5% male) with a median age of 76 years (interquartile range: 67–82). The study findings showed that the risk of stroke was highest within 5 days of experiencing a SCAF episode lasting 5.5 h or more in duration and diminished rapidly thereafter [14]. Therefore, based on the results of this study, it seems to be reasonable to start anticoagulant therapy when a SCAF lasts more than 5.5 h.

Conversely, despite ongoing research, the effectiveness of anticoagulant therapy in patients, particularly the elderly and frail, with AHREs lasting between 5 min and 5 h remains uncertain.

Overall, further research will be necessary to fully comprehend the exact threshold for detecting AHREs and the exact influence of AHRE duration on stroke risk.

## 3. Managing Subclinical Atrial Fibrillation in High-Risk Populations: Current Indications

Evidence suggests that SCAF is related to the volume of the left atrium. Results from the ASSERT II trial, which studied patients aged ≥65 with no prior AF diagnosis and a CHA_2_DS_2_-VASc score ≥ 2, reported a 34.4% SCAF detection rate over a follow-up period of 16.3 ± 3.8 months. However, this rate increased to 51.9% per year in individuals with a left atrial volume exceeding the median measurement of 73.5 mL [15].

The Implantable Loop Recorder to prevent Stroke in High-Risk Individuals (LOOP trial) involved 6004 patients aged 70–90 (average age of 74.7) with at least one other risk factor for stroke (such as high blood pressure, diabetes, past stroke, or heart failure) but no history of atrial fibrillation. Patients were randomly assigned in a 1:3 ratio to either implantable cardiac monitoring (ICM) or the standard of care. During a follow-up period of over 5 years, the intervention group had a significantly higher detection rate of AHREs (31.8%) compared to the control group (12.2%) [16]. The literature suggests that AF detection rates are significantly lower, ranging from 1–10% of the population, when using short-term or intermittent monitoring as opposed to implanted devices. The rates of arrhythmia detection were influenced by different levels of screening, types of devices used, and study populations when utilizing healthcare-professional-led techniques. Specifically, in the VITAL-AF [17], SCREEN-AF [18], and STROKESTOP [19] studies, the respective rates were 2%, 5%, and 12%. In combination, these data affirm that, among high-risk groups, CIEDs, and, particularly, loop recorder devices, have a strong sensitivity for detecting SCAF and are significantly more effective than short-term or intermittent monitoring.

The ACC/AHA 2023 guidelines [19] on diagnosis and management of AF state that an anticoagulation strategy is reasonable for patients with device-detected AHREs lasting ≥24 h and a CHA_2_DS_2_-VASc score ≥ 2 (recommendation class 2 A level B NR). It may also be reasonable in patients with device-detected AHREs lasting between 5 min and 24 h and a CHA_2_DS_2_-VASc score ≥ 3 (recommendation class 2 B level B NR). Patients with AHREs lasting less than 5 min should not be prescribed anticoagulant drugs unless there are other indications for anticoagulation (recommendation class 3 level B NR). These suggestions are classified as level B NR, indicating that they are based on moderate-quality evidence gathered from non-randomized studies. Therefore, randomized trials are desirable to better identify which patient with SCAF can take the highest net clinical benefit from anticoagulation. In the present review, we analyze the limitations of the CHA_2_DS_2_-VASc score which is currently used in clinical practice to identify SCAF patients who can be eligible for anticoagulation, and we suggest innovative approaches aimed to improve the patient selection that could be tested in future prospective studies. Specifically, there is a need to identify patients with SCAF at major risk of developing clinical AF and stroke and with a not-excessive hemorrhagic risk.

## 4. Do We Need New Scores for a Better Prediction of Stroke and Systemic Embolism in Elderly Patients with Subclinical Atrial Fibrillation beyond CHA_2_DS_2_-VASc Score?

### 4.1. The Impact of Comorbidities Not Considered in the CHA_2_DS_2_-VASc Score on the Risk of Stroke

According to our analysis, the reliability of the CHA_2_DS_2_-VASc score in accurately predicting the risk of stroke and systemic embolism in elderly and very elderly patients with SCAF is not satisfactory. This score in fact does not fully capture the complexity of the burden of comorbidities in older patients and also fails to consider significant factors such as obesity, chronic kidney disease (CKD), and obstructive sleep apnea syndrome (OSAS), which increase the risk of developing both clinical AF and stroke. Specifically, the presence of obesity can cause salt and water retention, leading to volume overload, arterial blood hypertension, and hyperactivity of the Renin–Angiotensin–Aldosterone System (RAAS). This sequence of events also triggers inflammation and oxidative stress, resulting in left atrial enlargement, local fibrosis in the heart tissue, and abnormal electrical conduction. These factors may contribute to clinical AF and promote its persistence [20,21,22].

Obesity not only increases the risk of developing AF, but it has been associated with an augmented risk of stroke in obese individuals compared with metabolically healthy normal-weight individuals (RR = 1.17, 95% CI: 1.11–1.23) in a meta-analysis of eight studies comprising 4,256,888 patients [23,24].

The probability of developing AF is significantly higher in individuals with CKD due to erythropoietin deficiency and the consequent anemia [25], hyperactivation of the RAAS [20], and an increased inflammatory state. Activation of cardiac fibroblasts by these mechanisms results in atrial cardiomyocyte fibrosis, causing left atrial dilation [26,27]. This leads to action potential shortening, depolarization of resting myocytes, and spontaneous phase 4 depolarization, ultimately increasing the risk of AF [28]. In addition, CKD results in electrolyte imbalances and elevated levels of urea and uremic toxins, such as indoxyl sulfate (IS), p-Cresol (PC), and p-Cresol sulfate (PCS). Cardiovascular remodeling and fibrosis can be promoted by the activation of neurohormones, oxidative stress, and inflammation, which can contribute to AF [29].

CKD is associated with a greater incidence of AF but has been also reported to be independently associated with an increased risk of stroke. Specifically, a recent multivariable Mendelian randomization analysis showed an independent effect of impaired kidney function, as assessed with estimated glomerular filtration rate, and ischemic stroke, mainly large artery stroke, even when controlled for systolic blood pressure [30].

Systemic hypoxemia, caused by both OSAS and COPD, can also trigger the development of AF [31,32]; furthermore, OSAS severity seems to be linked also to an increased risk of stroke [33]. Additionally, research suggests that gastrointestinal reflux disease (GERD) and reflux esophagitis (RE) disease may contribute to the onset of AF, although the specific underlying mechanism remains unclear [34]. RE independently increases the risk of stroke and TIA in hospitalized elderly patients with AF [35]. Moreover, GERD has been reported to be an independent causal risk factor for any stroke (odds ratio 1.19, 95% confidence interval 1.06–1.34) [36]. Similar findings were reported by another Mendelian randomization study [37].

Beyond the above-mentioned comorbidities, there are other relevant parameters which could modify the risk of stroke in patients with SCAF and that are not considered by scores used in clinical practice and suggested by guidelines. For instance, some objective factors that are not included in the CHA_2_DS_2_-VASc score and could impact the risk of stroke in patients with SCAF are (i) presence or absence of hypertrophic cardiomyopathy [38] and (ii) a comparison between poorly controlled and well-controlled hypertension, as well as (iii) the presence or absence of an enlarged left atrial volume (≥73 mL) or diameter (≥4.7 cm) [15,39]. Particularly, the left atrial enlargement is associated with both an increased risk of evolution towards clinical AF and an increased independent risk of stroke [40].

Based on these data, in our opinion, it is crucial to prospectively study novel scoring systems that consider comorbidities (i.e., obesity, CKD, OSAS, GERD, RE, and left atrial enlargement) in a large group of patients with SCAF. This will aid in accurately assessing the risk of ischemic events, individuating the subgroup of SCAF patients at major risk of developing clinical AF and/or stroke. However, it is important to underline that the exact contribution of each comorbidity in modifying the ischemic risk is very difficult to assess at the individual level; therefore, multiple efforts will be needed in this research field in the future. In recent years, some alternative scores to the CHA_2_DS_2_VASc score, for instance, ATRIA, have been proposed to estimate stroke risk in patients with AF and, in some cases, with encouraging results. ATRIA score, for example, has been reported to be more accurate than the CHA_2_DS_2_VASc score for identifying low-risk patients (Table 3). However, there are no sufficient data to support the use of these new scores instead of the CHA_2_DS_2_VASc score in SCAF patients in the view of selection of those subjects to be treated with anticoagulant therapy [41,42].

### 4.2. The Role of Frailty in Tailoring New Risk Scores for Elderly Individuals

The identification of new risk scores that are able to accurately stratify the prognostic outcomes of elderly patients with SCAF is of the utmost importance as there is currently a lack of evidence on this topic. With this in mind, we believe that a precise evaluation of frailty could support clinicians in deciding whether to start anticoagulation therapy for elderly individuals with SCAF.

In this scenario, frailty is defined as a clinical condition characterized by increased susceptibility due to the age-related decline in reserve and function across multiple physiological systems [43]. When combined with comorbidity and disability, it increases the likelihood of experiencing adverse health outcomes following minor stressors [44,45,46].

Frailty is a prevalent issue among older adults in high-income countries. Approximately 10% of individuals aged 65 and over living in the community are affected, and this number increases to 25–50% for those aged 85 and above. In acute care hospitals, frailty can affect up to 50% of patients aged 65 and above [47,48].

Various frailty tools have been developed to support with clinical assessments across different health and social care settings.

The Fried frailty phenotype (FP) is one of the most extensively validated and widely used methods for evaluating physical frailty. It is based on five criteria: unintentional weight loss, weakness or poor handgrip strength, self-reported exhaustion, slow walking speed, and low physical activity [49].

Frailty has been proven to have negative prognostic implications for elderly patients with AF, taking into account specific circumstances and evidence [50]. Up to 40% of AF patients were classified as frail in a systematic review involving 1,187,000 patients [51]. Additionally, frail AF patients faced an elevated risk of stroke, bleeding, and all-cause death [51,52]. Lastly, frailty is associated with longer hospitalization periods in AF patients [53].

Moreover, according to a recent prospective cohort study [54], multidimensional frailty, which measures vulnerability, showed a stronger correlation with a combination of all-cause mortality and rehospitalization within 1 year in older individuals with AF compared to physical frailty.

However, the potential impact of frailty on hemorrhagic risk in patients with AF remains a topic to debate. Some studies have suggested a link between frailty and an increased risk of bleeding in patients with AF [53,55,56]. A post hoc analysis was conducted on 20,867 participants from the ENGAGE AF-TIMI 48 trial, comparing two once-daily regimens of Edoxaban with warfarin. It was found that an increase in frailty correlated with a heightened risk of stroke and bleeding [57].

In contrast, other studies have failed to establish a connection between frailty and the risk of hemorrhage, either before or after commencing direct anticoagulant medication (DOACs) [53,58].

Recently, Soogard and colleagues performed a nationwide cohort study in Denmark to investigate the net clinical benefit of DOAC therapy in frail elderly patients with AF, taking into account both thromboembolic and hemorrhagic events. The results showed that the net clinical benefit was favorable (0.7%) in the whole cohort, but it tended to disappear with increasing age and frailty and was lowest among patients aged >75 years or with a high frailty level [59].

However, these findings need to be confirmed in other countries and may be influenced by the score used to assess frailty status. In addition to the FP criteria, several validated scales [60] are indeed available to diagnose frailty and its severity in older people, and it is still not clear which scale is the most accurate. The observed discrepancy in frailty burden among study populations and in methodologies used for assessment could account for the variation in estimated frailty prevalence in patients with AF, which ranged from 4.4 to 75% [61]. Therefore, further studies are warranted to establish a consistent method for evaluating frailty and assessing its predictive value in terms of both ischemic and hemorrhagic risk in the setting of AF. This will aid in the effective management of frail elderly patients with atrial arrhythmias, particularly those with SCAF. Indeed, in the context of SCAF, data on the impact of frailty on the risk of stroke are still lacking; in particular, sub-analyses from trials testing anticoagulant therapy in SCAF patients according to frailty status are not available so far.

## 5. Anticoagulation Therapy in the Elderly Patients with Atrial High-Rate Episodes Detected on Cardiac Implanted Electronic Devices: A Critical Issue

Anticoagulation therapy presents a unique challenge when considering elderly and very elderly patients due to their high risk for both ischemic and hemorrhagic events. However, the latest guidelines emphasize the importance of starting anticoagulation for those with clinical AF and a stroke risk higher than 1%, regardless of age and frailty, unless there are clear contraindications. There is ongoing debate regarding the appropriateness of administering anticoagulant therapy to individuals with SCAF, especially among those with a heightened risk of hemorrhaging and in advanced age.

The 2016 EHRA consensus document does not provide a clear threshold for the appropriate duration of AHREs, and there is also uncertainty about the correct method of stratifying patients with AHREs in terms of both ischemic and hemorrhagic risk.

A mentioned above, the 2023 ACC/AHA guidelines describe the level of evidence for anticoagulant therapy in SCAF patients as weak as it is not supported by randomized clinical trials.

In order to better clarify the risk/benefit profile of anticoagulant therapy in patients with SCAF, two randomized controlled trials were recently conducted: ARTESiA and NOAH-AFNET 6.

The ARTESiA (Apixaban for the Reduction of Thrombo-Embolism in Patients With Device-Detected Subclinical Atrial Fibrillation) trial was a randomized, double-blind, double-dummy study involving 4012 patients with SCAF, 36% of whom were women. The mean age of the patients was 76.8 ± 7.6 years, and the mean CHA_2_DS_2_-VASc score was 3.9 ± 1.1. The SCAF episodes lasted from 6 min to 24 h and were detected by CIEDs. The mean follow-up period was 3.5 ± 1.8 years. Enrolled patients were randomized 1:1 to receive either Apixaban 5 mg bid or 2.5 mg bid (when appropriate) or Aspirin 81 mg/die. The primary efficacy outcome, a composite of stroke and systemic embolism, was calculated in the intention-to-treat population. The primary safety outcome, major bleeding, was assessed in the on-treatment population.

The ARTESiA trial found that the Apixaban group had a 37% lower risk of systemic embolism or stroke compared to the Aspirin group, and a 49% lower risk of fatal or disabling stroke. However, the Apixaban group had a higher rate of major bleeding compared to the Aspirin group, even if Apixaban did not result in a higher risk of fatal bleeding than Aspirin [62].

In the NOAH-AFNET 6 [63] study, 2536 patients aged >65 years with implanted-cardiac-device-detected AHREs and at least one additional risk factor for stroke (at least one among age > 75 years, clinical heart failure, left ventricular ejection fraction < 45%, hypertension, diabetes, prior stroke or transient ischemic attack, or vascular disease) were randomly assigned to receive Edoxaban 60 mg/die or placebo/Aspirin as per clinical indication (around 50% of control subjects in this trial were treated with Aspirin). The median follow-up duration was 21 months. In this trial, an AHRE was defined as having >1 SCAF event at a rate > 180 atrial bpm for >6 min. The median duration of AHRE was 2.8 h, and the median CHA_2_DS_2_-VASc score was 4.

The trial found that anticoagulation therapy in the elderly patient with AHREs was not associated with a decreased incidence of cardiovascular death, stroke, or systemic embolism. However, it was associated with increased bleeding and all-cause mortality [63].

Thus, the NOAH-AFNET trial did not demonstrate any positive net clinical benefit of anticoagulation with Edoxaban in patients with SCAF.

In summary, the ARTESiA and NOAH-AFNET trials produced divergent results. ARTESiA suggested the possible efficacy of anticoagulation therapy in SCAF patients with an acceptable bleeding risk, while NOAH-AFNET did not endorse this treatment approach. However, there were differences between the two trials in several aspects. For instance, the participant pool was larger in ARTESiA, and different anticoagulants were administered (Apixaban versus Edoxaban). Additionally, the control group received different medications (Aspirin in 100% of control patients enrolled in ARTESiA versus placebo or Aspirin according to investigator’s preference in NOAH-AFNET) [64]. Furthermore, the inclusion criteria were quite different; for example, in NOAH-AFNET, the entry criterion regarding AHRE duration was simply SCAF lasting more than 6 min (without an upper limit of SCAF duration), while, in ARTESiA, only patients with SCAF lasting between 6 min and 24 h were recruited.

It is important also to take in mind that NOAH-AFNET was prematurely interrupted due to safety concerns; as a consequence, it could not be excluded that, if the trial had been continued until the achievement of the pre-defined sample size, Edoxaban could have been efficacious in reducing ischemic stroke risk. Finally, there is a relevant difference between these two trials in terms of primary end-point. Indeed, in NOAH-AFNET, the primary end-point was a composite of stroke, systemic embolism, and death from cardiovascular causes, while, in ARTESiA, only stroke and systemic embolism were considered as primary end-points (ignoring cardiovascular death). This is a crucial point because the effect of DOACs is expected to be relevant in preventing stroke and systemic embolism, while their role in reducing cardiovascular death is not expected to be equally important since cardiovascular death recognizes many causes and is influenced by several comorbidities (for instance, hypertension or diabetes), the majority of which are not directly influenced by anticoagulant therapy. All these aspects can easily explain the apparently conflicting results of the ARTESiA and NOAH-AFNET trials.

The meta-analysis of the two trials showed that anticoagulation therapy was effective in reducing the rate of ischemic stroke rate by approximately 32% (relative risk [RR] 0.68, 95% confidence intervals [CI] 0.50–0.92, high quality of evidence). The anticoagulation strategy was effective also in reducing a composite of cardiovascular death, all-cause stroke, peripheral arterial embolism, myocardial infarction, and pulmonary embolism. In contrast, DOAC increased major bleeding incidents by approximately 62% (RR 1.62, 95% CI 1.05–2.5, high quality of evidence) [65].

Although the meta-analysis produced clear results on a global scale, there is still significant uncertainty about which patient subgroups would benefit most from anticoagulation and when DOACs should be avoided due to an increased risk of significant bleeding. In this regard, a new score, called the DOAC score, was superior to the classical HAS-BLED score in assessing hemorrhagic risk with DOAC treatment [66]; therefore, it probably deserves to be incorporated in future studies aimed at improving the management of SCAF patients.

## 6. Conclusions and Future Directions

In summary, there is widespread agreement that the presence of SCAF in individuals with CIEDs may increase the risk of stroke or systemic embolism, especially in those with established stroke risk factors. Furthermore, SCAF may precede the onset of clinical AF. It is important to note that anticoagulant treatment can reduce the likelihood of stroke and systemic embolism in individuals with SCAF. However, it increases the risk of significant bleeding compared to either placebo or Aspirin.

Conversely, the appropriate duration threshold for clinically significant AHREs is unclear, and it is uncertain which specific group of patients with SCAF would benefit most from anticoagulant therapy. In an effort to enhance the classification of patients with SCAF, we propose the development of a new scoring system as an alternative to the routinely used clinical practice scores, such as the CHA_2_DS_2_-VASc score. In particular, we emphasize the need for further investigations aimed at improving the assessment of both ischemic and hemorrhagic risk. Regarding ischemic risk assessment, our proposal is to consider the possibility of validating a new scoring system assessing the impact of comorbidities such as CKD, obesity, OSAS, and GERD, as well as the success rate of blood pressure management (well-controlled vs. poorly controlled hypertension) and the presence of enlarged left atrial volume, and to compare the performance of this score with the CHA_2_DS_2_-VASc score in prediction of stroke risk in patients with SCAF. This theoretical score could potentially be improved by considering frailty. Nonetheless, the best method for diagnosing and measuring frailty remains uncertain. With respect to hemorrhagic risk, probably the DOAC score merits being tested to verify its usefulness in the context of management of SCAF patients.

Finally, there is insufficient evidence in the current literature to definitively recommend anticoagulant therapy for frail elderly patients with SCAF. In our estimation, it may be reasonable to administer anticoagulation therapy to non-frail elderly patients with acceptable bleeding risk according to DOAC score, especially in those with SCAF lasting more than 5.5 h. On the other hand, for frail and very severely frail elderly patients with SCAF and a poor overall prognosis, DOAC treatment may not be advisable. Undoubtedly, future studies may change this recommendation.

In conclusion, the management of SCAF in elderly patients remains a controversial matter. To gain a better understanding of this critical issue, future randomized controlled trials should include a wide cohort of older individuals, including those with severe frailty status.

## Figures and Tables

**Table 1:** Incidence of atrial fibrillation in elderly patients with implanted device.

Study and Year	Mean Age	Follow-Up	CHADSVASC Score (Mean)	Clinical Profile of Patients	Incidence of AF
RATE Registry, 2016	73.6 ± 11.8 for PPMs, 64.5 ± 12.6 for ICDs	22.9 months (median)	1.8 ± 1.0 for PPM 2.0 ± 0.8 for ICDs	All no permanent AF	45/300 (48%) of PPM patients and 155/300 (52%) of ICD patients of the representative samples studied
Healey et al., 2013	71.7 ± 14.4 for no AHRE 74.3 ± 13.7 for AHRE detected	Single center retrospective	2.02 ± 1.30 for no AHRE 2.23 ± 1.47 for AHRE detected	All	55.3% (246/445)
ASSERT, 2012	76 ± 7 for no AHRE 77 ± 7 for AHRE detected	2.5 years	2.3 ± 1.0 for no AHRE 2.2 ± 1.1 for AHRE detected	History of hypertension, no history of AF, no OAC use	34.7% (895/2580)
TRENDS, 2010	72.8 ± 9.9 for no AHRE 74.0 ± 9.1 for AHRE detected	1.4 years (mean)	4.1 ± 0.8 for no AHRE 4.2 ± 0.8 for AHRE detected	History of prior stroke, no history of AF, no OAC use, >1 stroke risk factor	28% (45/163)
BEATS, 2006		12 months, prospective		All	54% (137/254)
MOST, 2003	Median 73 (68.81) for no AHRE Median 75 (68.79) for AHRE detected	27 months (median)	NO	All	50% (156/312)
Gillis et al., 2002	70 ± 12	718 ± 383 days	NA	All	68% (157/231)

**Table 2:** Summary of studies on SCAF detected by CIEDs and thromboembolic risk.

Trial and Year	Number of Patients	Follow-Up Duration	AF Burden Threshold	Hazard Ratio for TE Event	TE Event Rate (Below vs. Above AF Burden Threshold
RATE Registry, 2016	5379 (3141 with pacemakers and 2238 with ICDs)	22.9 months (median)	Nonsustained atrial high-rate episodes with a duration from 3 atrial premature complexes to 15–20 s	0.87 (*p* = 0.51)	For nonsustained atrial high-rate episodes: 0.55% (0.34–0.76%) per year for pacemakers and 0.81% (0.50–1.12%) per year for ICDs
SOS-AF, 2014	10,016	2 years (median)	1 h	2.11 (*p* = 0.008)	0.39% per year overall
ASSERT, 2012	2580	2.5 years (mean)	6 min	2.5 (*p* = 0.007)	(0.69% vs. 1.69%)
Home Monitor CRT, 2012	560	370 days (median)	3.8 h	9.4 (*p* = 0.006)	2.0% overall
TRENDS, 2009	2486	1.4 years (mean)	5.5 h	2.2 (*p* = 0.060)	1.2% overall (1.1% vs. 2.4%)
Italian AT500 Registry, 2005	725	22 months (median)	24 h	3.1 (*p* = 0.044)	1.2% annual rate
Ancillary MOST, 2003	312	27 months (median)	5 min	6.7 (*p* = 0.020)	3.2% overall (1.3% vs. 5%)

**Table 3:** Two validated risk models for stroke score.

Risk Factors	ATRIA	CHADS-VASC
Age 65–74 y	3	1
Age ≥ 75 y	5	2
Age ≥ 85 y	6	
Hypertension		1
Female sex	1	1
Diabetes	1	1
Renal disease		1
Current smoking		
Congestive heart failure	1	1
Vascular disease		1
Previous stroke or TIA	2 (Age 75–84 y) 3 (Age > 85 y) 4 (Age 65–74 y) 8 (Age < 65 y)	2
Dementia		
Previous bleeding		
Proteinuria	1	
Low risk score	0–5	0
Intermediate risk score	6	1
High risk score	7–15	≥2
C-index	0.66	0.63
C-index	-	0.67
